# Is There an Association between a Tonsillar Diffuse Large B-Cell Lymphoma Arising after a Neck Squamous Cell Carcinoma of Occult Primary? A Case Report and Extensive Literature Review

**DOI:** 10.3390/hematolrep16020026

**Published:** 2024-04-29

**Authors:** Dimitris Tatsis, Athena Niakou, Konstantinos Paraskevopoulos, Stavroula Papadopoulou, Konstantinos Vahtsevanos

**Affiliations:** Department of Oral and Maxillofacial Surgery, Aristotle University, 57010 Thessaloniki, Greece; aeniakou@dent.auth.gr (A.N.); kostparas@yahoo.gr (K.P.); staupapadop@yahoo.gr (S.P.); vaxtseva@dent.auth.gr (K.V.)

**Keywords:** diffuse large B-cell lymphoma (DLBCL), tonsillar, squamous cell carcinoma (SCC)

## Abstract

Objectives: The aim of this review is to focus on the possibility of patients with squamous cell carcinoma to develop a second primary disease such as DLBCL, perhaps because of the irradiation of the head and neck area. Materials and methods: A case of an 89-year-old man is reported, who initially underwent surgical and complementary treatment for neck squamous cell carcinoma of occult primary and later for tonsillar diffuse large B-cell non-Hodgkin lymphoma. Results: The second primary was considered a recurrence in the neck of the original cancer of unknown primary, so a new surgical management was decided. The final pathology report described a diffuse large B-cell non-Hodgkin lymphoma. Conclusions: The importance of maintaining follow-ups for patients with occult primary cancers who are at an elevated risk of developing a metastasis or a second primary carcinoma outbreak is highlighted.

## 1. Introduction

Diffuse large B-cell lymphoma (DLBCL) is the most common subtype of non-Hodgkin lymphoma (NHL) worldwide, constituting up to 31% of these malignancies in adults [1]. Although the epidemiological data for DLBCL are limited, GLOBOCAN estimates 509,600 new cases of NHL were diagnosed in 2018, which comprise 2.8% of worldwide cancer diagnoses [2]. DLBCL is an aggressive NHL that promptly affects the B-lymphocytes, which are important part of the lymphatic system and make antibodies to fight infections. The occurrence of DLBCL increases with age, with most patients being at the age of seventy at the time of the diagnosis. However, this does not limit the appearance of this disease in children and young adults [3]. Despite decades of intensive research, DLBCL remains poorly understood. Predisposing factors that may affect the manifestation of this lymphoma include marginal zone lymphomas, immunodeficiency, previous chemotherapy, and infection from viruses such as Epstein–Barr (EBV), human immunodeficiency virus (HIV), *Helicobacter pylori* (*H. pylori*), and human T lymphotropic virus (HTLV) [4,5].

DLBCL has a wide clinical presentation, with symptoms varying from lymphadenopathy and hepatosplenomegaly to fever, weight loss, anorexia, abdominal pain, and cough. Symptoms are indicative to the Ann Arbor staging of the NHL [4]. The 5-year overall survival hovers at 73.2% and is related to the sex and the age. Most often, the treatment is a regimen of four drugs known as CHOP (cyclophosphamide, doxorubicin, vincristine, prednisone), plus the monoclonal antibody rituximab (Rituxan) and radiotherapy if the disease is localised [6].

The aim of this paper is to present a case of a patient who was treated in the Department of Oral and Maxillofacial Surgery at first for a neck squamous cell carcinoma of occult primary and 4.5 years later for a tonsillar DLBCL non-Hodgkin lymphoma. 

## 2. Case Description

An 89-year-old male patient presented in 2015 with a persistent firm lump on the left side of his neck, extended at the submandibular triangle for over 4 months, sizing 3.2 × 2.9 cm. The patient stated in his medical history to have hypertension under treatment, but except that, he did not have any other medical condition. No tobacco use was recorded. On physical intraoral examination, everything was normal, and no masses were located. Panendoscopy and bronchoscopy did not show any signs of malignancy. Fine needle aspiration (FNA) biopsy was taken from the lump, which was consistent with squamous cell carcinoma (SCC) of moderate differentiation. 

**Figure 1:**
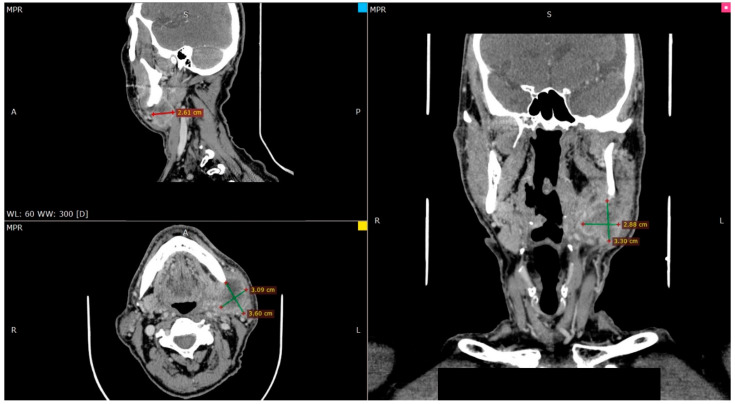
CT imaging of the initial neck mass. A: anterior, P: posterior, R: right, L: left, S: superior.

Cranial and neck CT (Figure 1) and PET scan examination were carried out and the results showed a large, very hypermetabolic lymphadenopathy at the anatomical position of the deep lobe of the left parotid gland and extending to the left submandibular region (focusing on level IA on the left and extension within the boundaries of level IIB as well as IB anteriorly where two distinct hypermetabolic lymph nodes were observed). These findings were in line with malignant metastatic lymph node disease. No obvious pathological findings were observed in the structures of the neck indicative of a possible primary focus.

The lump was excised under general anaesthesia. The patient underwent a modified radical neck dissection type I surgery (MRND), with concomitant reverse marginal left mandibulectomy to manage the bone infiltration, partial excision of the corresponding masseter and the medial pterygoid muscle and a large part of the parotid gland. Postoperative course was uneventful, and the patient was discharged on the seventh postoperative day.

The pathology report confirmed the development of squamous cell carcinoma. The neoplasm had varying cell differentiation, but mostly had poor differentiated squamous cells. Immunohistochemical testing in the low differentiation region demonstrated the expected immunophenotype p63, Ck 5/6. The neoplastic cells infiltrated the tissues adjacent to the salivary glands (parotid-submandibular) while extending between the lobes of the parotid gland. The tumour also significantly infiltrated the lower jawbone (periosteum) and skeletal muscle at a location near the parotid gland. The 27 lymph nodes evaluated were disease free. Summarizing the histological and immunohistochemical findings, the diagnosis was compatible with keratinizing neck squamous cell carcinoma of good to low differentiation without lymph node metastases (0/27) (ICDO: 8071/3). 

The postoperative treatment included photon radiotherapy and the patient received 6400 cGy with the technique of multiple head and neck fields, in 32 fractions over 6 weeks. The patient was scheduled for timely follow-ups.

**Figure 2:**
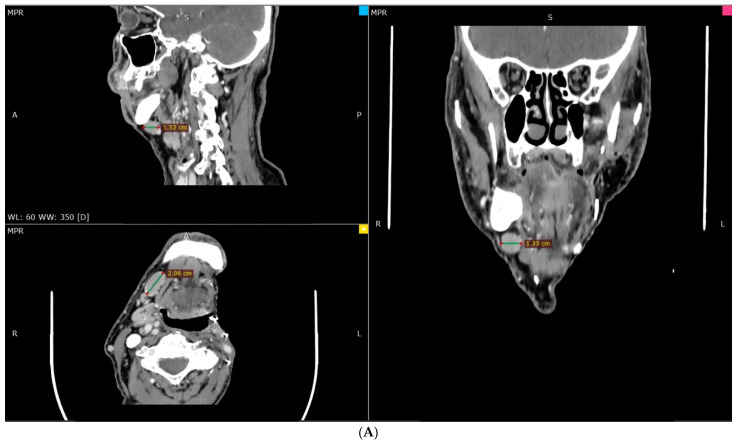

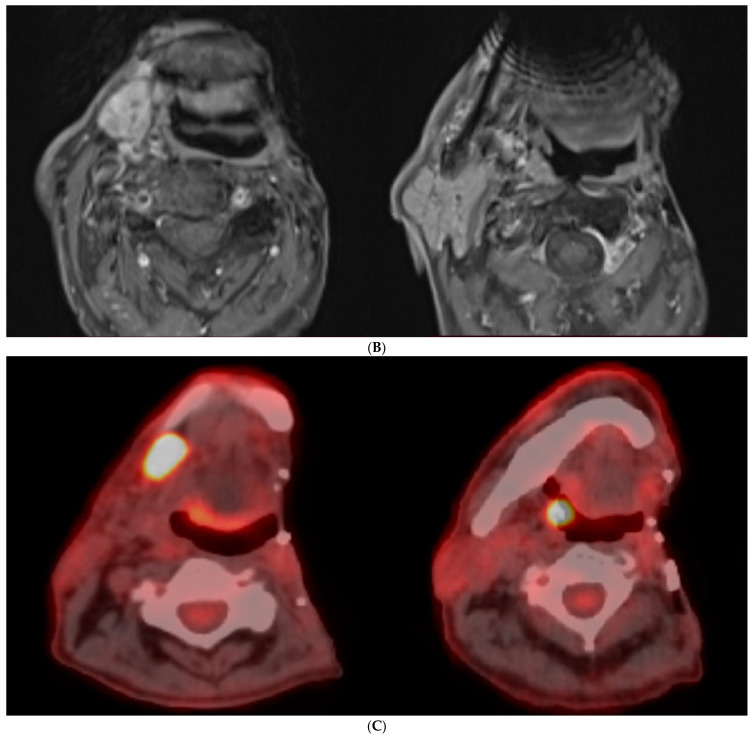
(**A**) CT, (**B**) MRI, and (**C**) PET-CT of the second primary disease on the contralateral neck. (A: anterior, P: posterior, R: right, L: left, S: superior.) The avidity in the image 2C depicts the two areas where the disease is evident in the PET-CT.

Four and a half years after the initial diagnose and excision, the patient underwent PET/CT/MRI on the suspicion of disease relapse on the right side of the neck (Figure 2). The physical intraoral and panendoscopy examination showed no malignant findings. However, MRI and CT of the visceral skull showed a pathologically enlarged submandibular lymph node on the right side, suspected of recurrence. There was also a finding at the height of the right parietal tonsil. Due to the high suspicion of recurrence of the initial, locally advanced, disease, a modified neck lymph node radical clearance of levels IB, II, III, and V (MRND type III) was decided and performed, along with a right tonsillectomy.

The microscopic findings for the right tonsillar showed a malignant neoplastic process with a lymphoid character. Focal ulcers and necrosis of the superficial multi-layered squamous epithelium were observed. 

**Figure 3:**
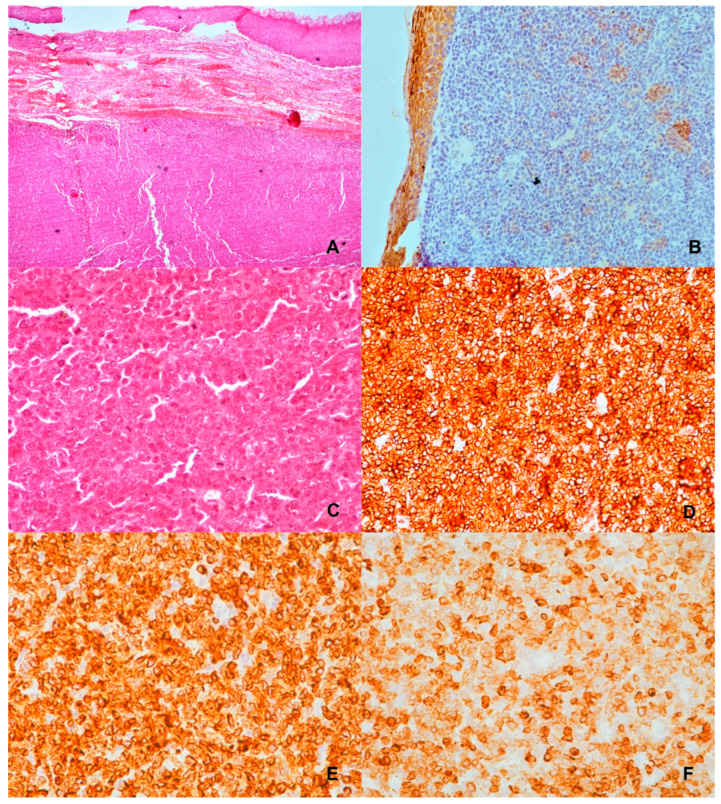
Histological examination of the second primary disease (tonsillar tissue). (**A**) Squamous cells without dysplasia, malignant neoplastic process with lymphoid character, H/E ×100. (**B**) CK5/6, no dysplasia evident, ×200. (**C**) Lymphoid characters with multiple forms and nuclear lesions, H/E ×400. (**D**) CD20, ×400. B-cell orientation, large lymphoid cells are observed, ×400. (**E**) CD79a, ×400. (**F**) BCL-2 stain, ×400.

The immunophenotype of tumour population was CD20+, CD79a+, Pax-5+, CD3− CD5-CD23-, ALK-, EBV-, CD30-, Cyclin D1-. The Hans cell origin algorithm was MUM1+ BCL-6+ CD10+ (GCB subtype) and the cell cycle regulatory proteins were Bcl-2 + C-myc-. The cell proliferation index was Ki-67~85% (Figure 3).

In total, 7 out of the 32 excised lymph nodes had malignant lesions, all in the cervical level II. 

The final histological and immunohistochemical findings were compatible with non-Hodgkin B lymphoma of cellular origin diffuse type from large cells not further identified (DLBCL-NOS/WHO 2017). After the DLBCL diagnosis, the patient was informed and referred to the Haematology department for further assessment and definitive treatment. The patient received four cycles of R-CVP (rituximab, cyclophosphamide, vincristine sulphate, and prednisone) due to his advanced age. One year later, he remains disease-free.

## 3. Discussion

The occurrence of different metachronous malignancies in the same patient is a rare event and emerges as a combination of multiple factors such as the site and the type of the tumour, the age of the patient, and other environmental factors. The pathogenetic mechanism in this situation may be complex and the course of the treatment difficult [7]. A tonsillar diffuse large B-cell lymphoma (DLBCL) arising after a head and neck squamous cell carcinoma (SCC) of occult primary has been scarcely reported in the medical literature until today; hence, we publish this case. 

Second malignancies have been described in the literature. Tezer et al. [8] reported an epiglottic SCC in a 62-year-old man who underwent total laryngectomy and neck dissection, and in the neck specimen, lymph nodes affected by a B-cell lymphoma were diagnosed. Further imaging revealed a nasopharyngeal mass of a B-cell high-grade malignant lymphoma. Hubermann et al. [9] reported a similar-aged patient with simultaneous diagnosis of metastatic SCC and T-cell NHL in the same cervical lymph node. Similarly, a patient with concurrent oropharyngeal SCC and nasopharyngeal malignant lymphoma was reported by Watanabe et al. [10]. Kader et al. [11] identified eight patients with low-grade lymphoma and metastatic lymph nodes in the head area from primary cutaneous SCC, highlighting the diagnostic challenge. Millwaters et al. [12] made note of the diagnostic difficulty in a patient who was initially treated for malignant lymphoma but persistent cervical lymphadenopathy revealed the second primary SCC. A 71-year-old patient was reported with, apart from primary larynx SCC and NHL in the cervical lymph nodes, papillary thyroid carcinoma [13]. Another patient with triple primary laryngeal SCC, NHL, and Kaposi sarcoma was reported in reference [14]. A small case series reported that secondary SCC after NHL presents with a poorer prognosis [15]. In addition, another case series hypothesized that radiotherapy for NHL in the head and neck area can induce SCC as a late effect [16]. Finally, Thakur et al., reported an atypical location of a primary NHL in the infratemporal fossa of a 41-year-old patient who developed oral SCC approximately 2 years after initial diagnosis [17]. It is quite evident from the literature presented that the opposite manifestation is quite rare, compared to the aforementioned, in that NHL after an SCC of the oral cavity is extremely rare. It is known that some cancers may be radiation induced, but the latency is expected to be significantly higher, and from the presented cases, it is established that simultaneous disease was evident in the majority of them [18]. Table 1 summarizes the aforementioned cases.

Different infectious agents, such as Epstein–Barr virus (EBV), human T-cell leukaemia virus (HTLV), and others, are associated with NHLs [19,20]. Hereditary immunodeficiency disorders and autoimmune disorders are also significant predisposing factors of an NHL occurrence. Furthermore, immunosuppressants and chemotherapeutic agents or exposure to environmental factors are potentially significant in the aetiology of NHL, although the impact of these chemicals has not yet been fully elucidated [21]. Thus, the exact causes of the increased cases of NHL are not fully understood but could be related to exposure to the risk factors described above [22].

Although the correlation between chemo-/radiotherapy and NHLs is difficult to confirm, it has raised fundamental questions specifically about the development of secondary cancers. Radiotherapy causes single- and double-strand DNA breaks which sometimes lead to synchronous or metachronous carcinogenicity [23]. The patient presented in our case report was initially treated for a head and neck squamous cell carcinoma (SCC) of occult primary with surgery and postoperative radiotherapy and after a 4 and a half-year follow-up he developed a mass which turned out to be DLBCL. The question arising is whether radiation or chemotherapy used to treat a primary tumour can be a causative factor for another secondary cancer to emerge. More cases and extensive analyses should be reported in the literature to formulate a specific relationship between radiation and NHL outbreak.

Furthermore, every patient with a head and neck tumour requires meticulous clinical examination and workup prior to any intervention. SCC is the most common malignancy of the head and neck, but not the only one, as differential diagnoses of a head and neck tumour or cervical lymphadenopathy should include, among others, lymphoma [24]. Different treatment modalities should be implemented, depending on the diagnosis [25,26]. The prognosis is also different, as even different subtypes of lymphomas have different outcomes. According to a report conducted among 110 patients, extranodal DLBCL showed significantly better disease-specific survival in comparison with nodal head and neck lymphomas (90% versus 60% in 5 years, *p* = 0.011) [27]. Another research study indicated that MALT lymphomas have a better prognosis compared with DLBCLs (5-year survival rate of about 90% compared to 50%, respectively) [28]. The prognosis is also based on the Ann Arbor staging system of the NHL, where malignant lymphomas are staged due to the involvement of the lymph nodes affected [6]. 

To the best of our knowledge, tonsil DLBCL develops a malignant behaviour and occurs in patients in any age, but the majority of cases occur in elderly patients [29]. A study published by Zhang et al., suggests that a good long-term prognosis can be achieved without treating patients with primary tonsil DLBCL of initial stages (stage I and II of Ann Arbor staging) with chemo/radio therapy but only with tonsillectomy and close and regular follow-ups [30]. However, the participants in this study were only children and young adults, so further research is required to reach to a conclusion and determine whether primary DLBCL can be remain untreated. The patient presented in this report received four cycles of R-CVP, instead of the standard regime of six cycles of R-CHOP (cyclophosphamide, doxorubicin, prednisone, rituximab, and vincristine). Advance age and previous malignancy were prohibiting factors for R-CHOP.

Very few cases have been published showing the correlation between SCC or other types of cancer and DLBCL. Fonseca et al., present a case of a patient having a lung squamous cell carcinoma and DLBCL of ileum synchronously. The patient was an alcoholic and a smoker, which are risk factors for recurrent cancer, but the relationship between genetic perturbations and other environmental pathogenetic factors and carcinogenicity, as mentioned before, remains unclear [31]. 

Inferentially, about 20% of patients with head and neck cancer have distant metastases or discover a secondary primary cancer during a follow-up PET scan [32]. DLBCL is found to be correlated with SCC and with numerous predisposed genetic and environmental factors and, in our case, with the previous radiotherapy used to treat the SCC. Extensive research must be conducted to formulate a definite association between them. 

## 4. Conclusions

The presence of a second primary non-Hodgkin lymphoma in the head and neck region subsequent to the effective treatment of an initial squamous cell carcinoma might potentially be linked to the administration of postoperative radiotherapy, but the exact mechanism remains unknown. It is crucial for patients to persist with their follow-ups to detect a malignancy promptly and start the appropriate treatment as soon as possible to improve the treatment’s results.

## Figures and Tables

**Table 1:** Second malignancies of head and neck SCC and non-Hodgkin lymphoma.

Case	Age (in Years)	Gender	Squamous Cell Carcinoma (SCC) Diagnosis	Lymph Node Involvement in Neck	Lymphoma Diagnosis	Time Lapse between the 2 Diagnoses
Habermann et al., 1997 [9]	64	Male	Left tonsillar SCC	yes	T-cell NHL, lymph nodes	Simultaneously
Watanabe et al., 2007 [10]	57	Male	oropharyngeal SCC, cT2N2bM0	yes	Nasopharyngeal ML, stage IE	Simultaneously
Kader et al., 2016 [11]	8 patients, mean age 79.5	-	Skin SCC (patients with oral SCC excluded from study)	yes	Low-grade non-Hodgkin lymphoma or chronic lymphoid leukaemia	Mean time 3.4 years, lymphoma first
Millwaters et al., 2008 [12]	70	Male	Left tongue base SCC	yes	Follicular non-Hodgkin lymphoma	Simultaneously
Sigh et al., 2016 [13]	71	Male	Laryngeal SCC (and papillary thyroid carcinoma)	yes	Nodal marginal zone lymphoma	Simultaneously
Yildirim et al., 2019 [14]	74	Male	Laryngeal SCC	yes (and Kaposi sarcoma in cervical lymph node)	Non-Hodgkin lymphoma in tongue base	Simultaneously
Li et al., 2020 [15]	4 patients, mean age 56.5	Male	Metachronous head and neck SCC	yes	Non-Hodgkin lymphoma or chronic lymphocytic leukaemia	1–4 years, lymphoma first
Toda et al., 2009 [16]	4 patients, mean age 53	3 Male, 1 Female	Metachronous tongue, gum and maxillary SCC	unknown	Non-Hodgkin lymphoma	8.7–22.7 years, lymphoma first
Thakur et al., 2009 [17]	41	Female	Metachronous right retromolar SCC	Unknown	Non-Hodgkin lymphoma, right infratemporal fossa	1 year, lymphoma first

## Data Availability

The original contributions presented in the study are included in the article, further inquiries can be directed to the corresponding author.
